# Synaptic and Non‐Synaptic Functions of PTPRD: A Receptor Tyrosine Phosphatase at the Crossroads of Neural Circuitry and Metabolism

**DOI:** 10.1111/jnc.70292

**Published:** 2025-11-10

**Authors:** Seoyeong Kim, Jae Jin Shin, Muwon Kang, Yunho Yi, Eunjoon Kim

**Affiliations:** ^1^ Department of Biological Sciences Korea Advanced Institute for Science and Technology (KAIST) Daejeon Korea; ^2^ Center for Synaptic Brain Dysfunctions Institute for Basic Science (IBS) Daejeon Korea

**Keywords:** Neuropsychiatric and metabolic disorders, PTPRD, synaptic adhesion molecule

## Abstract

Protein‐tyrosine phosphatase receptor‐type D (PTPRD) is an adhesion‐coupled phosphatase that translates extracellular binding codes into intracellular phosphotyrosine signaling from embryogenesis through adulthood. Alternative inclusion of the Ig‐domain mini‐exons meA and meB tailors the ectodomain surface, thereby dictating high‐affinity engagement with IL1RAPL1, IL1RAP, Slitrks, LRFN4/5 (SALM3/5), neuroligin‐3, and other postsynaptic partners. Intracellularly, the catalytically active D1 domain and scaffold‐like D2 module, anchored to liprin‐α, coordinate presynaptic vesicle release, postsynaptic receptor composition, and synaptic plasticity. Beyond synapses, PTPRD restrains embryonic neurogenesis, promotes STAT3‐dependent gliogenesis, accelerates oligodendrocyte myelination, and guides Sema3a/Fyn‐mediated axon and dendrite patterning. In the adult brain it serves as the high‐affinity hypothalamic and cerebellar receptor for asprosin, thereby coupling systemic energy and hydration states to feeding and drinking behavior. Human genetic studies and mouse models link these molecular activities to a spectrum of conditions—including restless legs syndrome, addiction, Alzheimer's disease, ADHD, OCD, autism spectrum disorder, and metabolic syndrome. Because PTPRD functions are pathway‐specific and shaped by mini‐exon usage or redundancy with other family members (PTPRS/PTPRF), domain‐ or ligand‐selective interventions represent plausible therapeutic strategies. Elucidating its full ligand repertoire, substrate landscape, and structural basis for allosteric regulation will be critical for converting this versatile receptor from a mechanistic curiosity into a tractable target for neurodevelopmental, neuropsychiatric, and metabolic disorders.

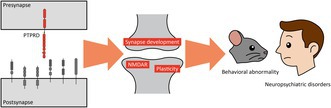

## Graphical abstract

PTPRD (receptor‐type protein tyrosine phosphatase delta) translates extracellular adhesion “codes” into intracellular phosphotyrosine signaling that shapes brain circuits. Alternative inclusion of micro‐exons meA and meB sculpts PTPRD's Ig domain, dictating trans‐synaptic binding to partners such as IL1RAPL1 or IL1RAP and thereby tuning synapse development, NMDAR function, and plasticity. Beyond synapses, PTPRD serves as the neural receptor for the fasting hormone asprosin, linking systemic metabolic state to feeding and thirst. By integrating adhesion and signaling, PTPRD emerges as a therapeutic entry point for neurodevelopmental, neuropsychiatric, and metabolic disorders.

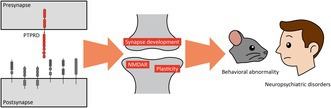

**Abbreviations**
3cKO/6cKO: triple/sextuple conditional knockout
AAV: adeno‐associated virus
ADAM10: a disintegrin and metalloproteinase 10
ADHD: attention‐deficit/hyperactivity disorder
AgRP: agouti‐related peptide
ALDH1L1: aldehyde dehydrogenase 1 family member L1 (marker of astrocytes)
AMPAR: AMPA‐type glutamate receptor
AP: action potential (context‐dependent)
AP2B1: adaptor protein 2 subunit β1 (β‐adaptin)
ARH: arcuate nucleus of the hypothalamus
ASD: autism spectrum disorder
AURKA: aurora kinase A
BBB: blood–brain barrier
BDNF/TrkB: brain‐derived neurotrophic factor/TrkB receptor
CA1/CA2/CA3: hippocampal cornu ammonis fields
CASK: CaMKII‐associated serine/threonine kinase
CC1: APC/CC1; marker of mature oligodendrocytes
CF: climbing fiber
cKO: conditional knockout
CNS: central nervous system
CNV: copy‐number variant
CPP: conditioned place preference
Cre: Cre recombinase
CRMP2: collapsin response mediator protein 2
cTKO: conditional triple knockout
D1/D2 (PTPRD): catalytic (D1) and pseudo‐phosphatase (D2) domains
DCN: deep cerebellar nuclei
DG: dentate gyrus
DIO: diet‐induced obesity
DRG: dorsal root ganglion
EAS: East Asian ancestry
EC: entorhinal cortex
eEPSC: evoked excitatory postsynaptic current
EM: electron microscopy
Emx1‐Cre: telencephalon‐restricted Cre driver
EPM: elevated plus‐maze
EPSC: excitatory postsynaptic current
EUR: European ancestry
fl/fl: homozygous floxed allele
FNIII: fibronectin type III domain
Fyn: Src‐family tyrosine kinase Fyn
GABAR: γ‐aminobutyric acid receptor
GAD: glutamate decarboxylase (marker of GABAergic terminals)
GC: granule cell (dentate)
GFAP: glial fibrillary acidic protein
GWAS: genome‐wide association study
ICD: intracellular domain (after γ‐secretase cleavage)
Ig: immunoglobulin‐like domain
IHC: immunohistochemistry
IL1RAP: interleukin‐1 receptor accessory protein
IL1RAPL1: interleukin‐1 receptor accessory protein‐like 1
IN: interneuron
JAK: Janus kinase
KO: knockout
Kv1.4/KCNA4: voltage‐gated potassium channel subunit
L7 (Pcp2): Purkinje cell protein 2 (Purkinje cell–specific Cre driver)
LAR‐RPTPs: type IIa receptor protein tyrosine phosphatases (PTPRF/LAR, PTPRD, PTPRS)
LD: light–dark box
LRFN4/5 (SALM3/5): leucine‐rich repeat and FNIII domain–containing proteins 4/5
LRR: leucine‐rich repeat
LRRC4B (NGL‐3): leucine‐rich repeat–containing 4B (netrin‐G ligand‐3)
LT (lamina terminalis): SFO/OVLT/MnPO complex
LTP: long‐term potentiation
mAb/mAbs: monoclonal antibody/antibodies
MAP 2: microtubule‐associated protein 2
meA: PTPRD mini‐exon A
meA/meB: PTPRD mini‐exons A/B
MEC: medial entorhinal cortex
MEK–ERK: MAPK kinase–extracellular signal‐regulated kinase pathway
mEPSC/mIPSC: miniature EPSC/IPSC
MnPO: median preoptic nucleus
mPFC: medial prefrontal cortex
NGL‐1/NGL‐3: netrin‐G ligands 1/3
NLGN3: neuroligin‐3
NMDAR: NMDA‐type glutamate receptor
NMDAR/AMPAR: NMDA‐/AMPA‐type glutamate receptors
non‐REM: non‐rapid eye movement sleep
NPY: neuropeptide Y
Nrxn: neurexin
OC: obsessive‐compulsive (traits)
OCD: obsessive‐compulsive disorder
oEPSC/oIPSC: optogenetically evoked EPSC/IPSC
OFT: open‐field test
OR: odds ratio
OVLT: organum vasculosum of the lamina terminalis
PBN: parabrachial nucleus
PC: Purkinje cell
Pcp2/L7: Purkinje cell protein 2 (L7)
PDB: Protein Data Bank
PDGFRβ: platelet‐derived growth factor receptor‐β
PF: parallel fiber
PPI: prepulse inhibition
PPR: paired‐pulse ratio
PPR/PPD: paired‐pulse ratio/paired‐pulse depression
PSD: postsynaptic density
PTPRD: protein tyrosine phosphatase receptor type D
Ptprd^−/−^
: global Ptprd knockout
Ptprd^fl/fl^
: homozygous floxed Ptprd allele
PTPRF (LAR): protein tyrosine phosphatase receptor type F
PTPRS: protein tyrosine phosphatase receptor type S
pTyr: phosphotyrosine
RIM1: Rab3‐interacting molecule 1
RLS: restless legs syndrome
RMP: resting membrane potential
RNAi/siRNA/shRNA: RNA interference/small hairpin RNA
SALM: synaptic adhesion‐like molecule
SAM (Liprin‐α): sterile α motif (liprin‐α)
SC: Schaffer collateral
Sema3A: semaphorin 3A
SFO: subfornical organ
SK channel: small‐conductance Ca^2+^‐activated potassium channel
Slitrk1–6: SLIT and NTRK‐like family proteins 1–6
SNP: single‐nucleotide polymorphism
SO/SR/SLM: stratum oriens/radiatum/lacunosum‐moleculare
SPR: surface plasmon resonance
STAT3: signal transducer and activator of transcription‐3
SuB: subiculum
SYNJ1: synaptojanin‐1
SYT1: synaptotagmin‐1
tdTomato: tandem dimer Tomato fluorescent reporter
TKO: triple knockout
vGluT2: vesicular glutamate transporter 2
WT: wild‐type

## 1 Introduction

The construction of a functional vertebrate brain depends on seamless crosstalk between extracellular adhesion events, which position neurons and align pre‐ and postsynaptic membranes, and intracellular signaling cascades, which refine those contacts by modulating the activity of synaptic proteins. Receptor protein tyrosine phosphatases of the LAR family (LAR‐RPTPs), containing PTPRD (human and mice; also known as PTPδ), PTPRS (PTP*σ*), and PTPRF (LAR), embody this principle: their ectodomains form selective trans‐synaptic complexes with various ligands, while their cytoplasmic tails harbor a catalytically active D1 phosphatase and a scaffold‐like D2 pseudo‐phosphatase that anchors presynaptic active‐zone proteins. Alternative splicing at two short “mini‐exons” further customizes each paralogue's binding repertoire, enabling cell‐type and synapse‐specific control over whether a nascent bouton will become mature presynaptic nerve terminals (see the “Domain structure and functions of PTPRD” section below for details).

Among the LAR family proteins, PTPRD has risen to prominence over the past two decades, propelled by convergent human‐genetic and mechanistic data. Genome‐wide association studies highlighted *PTPRD* as a major susceptibility locus for restless‐legs syndrome and have since extended its reach to addiction, obsessive‐compulsive disorder, attention‐deficit/hyperactivity disorder, autism spectrum disorder, anxiety, and Alzheimer disease (see the “PTPRD mutations in brain disorders” section below for details). Concordantly, constitutive or circuit‐restricted *Ptprd* mutations in mice alter dendritic architecture, synapse number and function, sleep architecture, reward processing, executive control, social interaction, and age‐related cognition. At the cellular level, PTPRD not only assembles synapses but also guides axons, sculpts dendrites, regulates the neuro‐to‐glio‐genic switch, and accelerates early myelination, underscoring its influence at virtually every stage of circuit formation.

Details of PTPRD and the other two LAR‐PTPRs (PTPRS and PTPRF) and their trans‐synaptic interactions and functions have been comprehensively reviewed elsewhere (Uhl and Martinez 2019; Cornejo et al. 2021; Um and Ko 2013; Kim et al. 2021; Won et al. 2019; Takahashi and Craig 2013; Lie et al. 2018; Stoker 2015; Chagnon et al. 2004). Building on that foundation, the present article integrates the newest mechanistic insights from *Ptprd*‐mutant mice and complementary structural, biochemical and phospho‐proteomic studies. After revisiting PTPRD's domain architecture and splice‐code‐dependent ligand selectivity, we discuss in vivo evidence for its roles in synaptic density, transmission and plasticity, as well as non‐synaptic processes such as axon guidance, neuron–glia fate switching, and myelination. We also highlight emerging metabolic functions of *PTPRD* in feeding and thirst regulation. Finally, we outline outstanding questions, including the identification of synaptic phospho‐substrates of PTPRD and strategies for modulating its functions across neurodevelopmental, neuropsychiatric, and metabolic disorders.

## 2 Domain Structure and Functions of PTPRD

**FIGURE 1:**
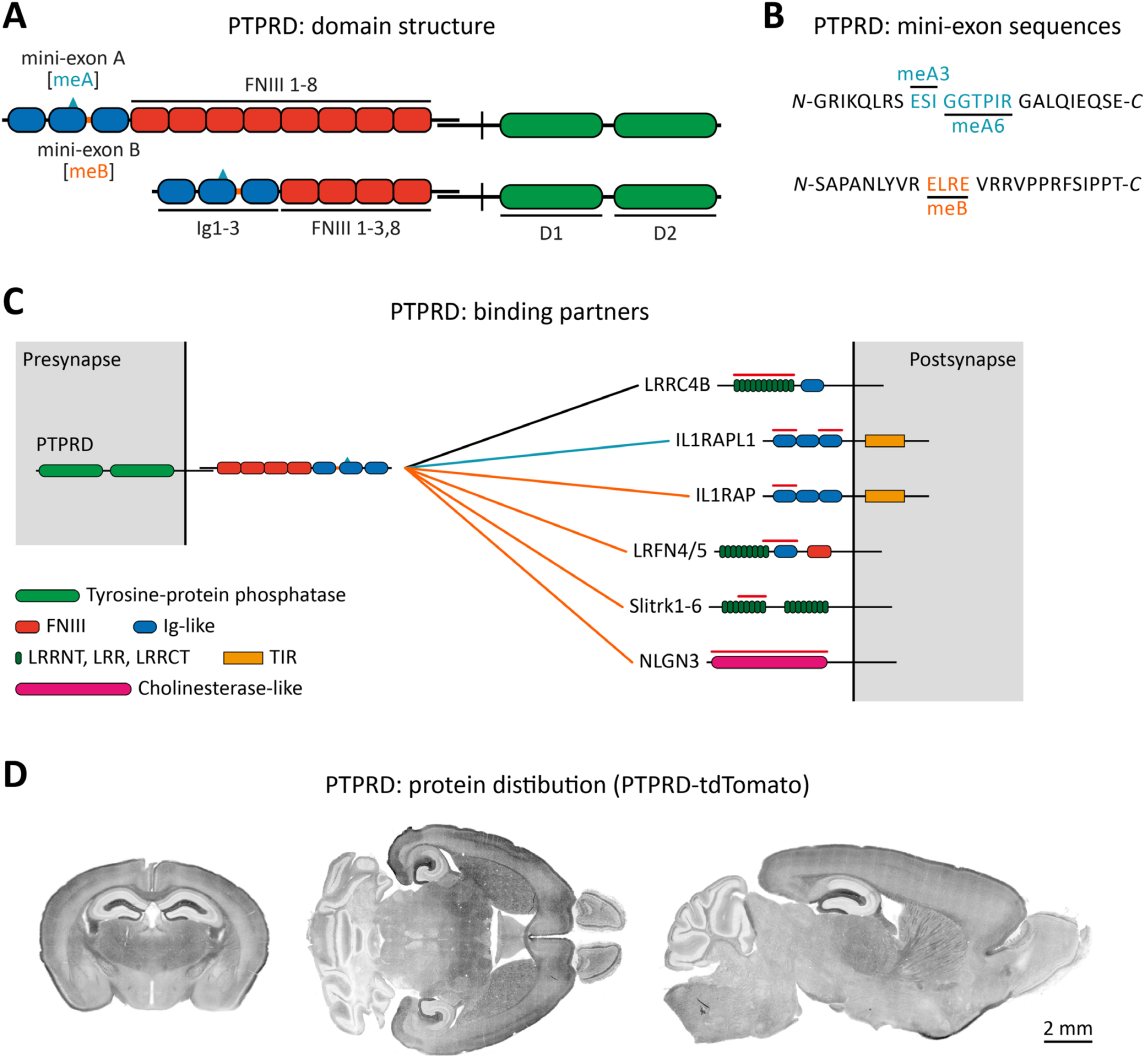
Domain structure, splice variants, protein interactions, and distribution patterns of PTPRD mRNA and protein. (A) Domain structure and alternative splicing in PTPRD. Schematic of the full‐length extracellular region (Ig‐like domains; fibronectin type III, FNIII) and tandem intracellular phosphatase domains (D1, D2). Two mutually independent mini‐exons are subject to alternative splicing; mini‐exon A (meA) within the second Ig domain and mini‐exon B (meB) immediately N‐terminal to the third Ig domain. A common splice isoform lacking FNIII4‐7 is shown beneath for comparison. (B) Amino acid sequences of mini‐exon A and B of PTPRD. MeA located in Ig2‐domain consists of two mini‐exons meA3 (3 amino acids) and meA6 (6 amino acids). MeB adds a 4‐residue extension to the Ig2‐Ig3 hinge. (C) Splice‐code‐dependent trans‐synaptic interactions of PTPRD. A presynaptic PTPRD molecule (left) engages multiple postsynaptic adhesion partners (right). Colored lines indicate splice requirements determined by structural and biochemical studies: Black, interaction with NGL‐3/LRRC4B involves the FNIII domains of PTPRD and is independent of meA and meB; cyan, inclusion of meA is required for high‐affinity binding to IL1RAPL1; orange, meB‐dependent interactions where meB inclusion enables binding to IL1RAP, Slitrk1–6, and SALM3/5 (LRFN4/5), whereas meB exclusion facilitates binding to neuroligin‐3 (NLGN3). (D) Distribution patterns of PTPRD proteins. Low‐magnification coronal, horizontal, and sagittal brain sections from the PTPRD‐tdTomato reporter mice reveal robust protein expression across several brain regions, including the cerebral cortex, hippocampus (specifically the CA1 stratum lacunosum‐moleculare and the dentate gyrus molecular layer), thalamus, and corpus callosum. These images have been reported previously (Park et al. 2020). Scale bar, 2 mm.

PTPRD is a hybrid transmembrane protein containing extracellular cell adhesion domains and intracellular tyrosine phosphatase domains (Figure 1A). Its extracellular region contains three immunoglobulin (Ig)‐like domains and eight fibronectin type III (FNIII) domains, followed by a single transmembrane domain and two intracellular phosphatase domains, which regulate various extracellular adhesions and intracellular interactions (summarized in Table 1). The membrane‐proximal phosphatase domain (D1) is catalytically active, whereas the distal domain (D2) lacks enzymatic activity and instead mediates interactions with various presynaptic proteins. Alternative splicing can omit four of the eight FNIII domains (FNIII4–7), although the precise functional consequences of these variants remain poorly understood.

**TABLE 1:** PTPRD's trans‐synaptic interaction partners, domains, and splice requirements.

Partner	PTPRD domain	Partner domain	PTPRD mini‐exon dependency	Evidence	References
LRRC4B (NGL‐3)	FNIII1–2	LRR	None	Cell surface binding; synaptogenesis assays	(Kwon et al. 2010; Woo, Kwon, Choi, et al. 2009)
IL1RAPL1	Ig1–3	Ig1 and Ig3	meA and partially meB	Crystal structures (4YH7); Surface plasmon resonance (SPR); cell surface binding; synaptogenesis assay	(Yamagata, Yoshida, et al. 2015; Yoshida et al. 2011; Park et al. 2020)
IL1RAP	Ig2–3	Ig1	meB and partially meA	Crystal structures (4YFD); SPR; cell surface binding; synaptogenesis assay; total proteomics	(Yamagata, Yoshida, et al. 2015; Yoshida et al. 2012; Kim et al. 2025)
LRFN4/5 (SALM3/5)	Ig2–3	LRR and Ig	meB	Crystal structures (5XNP); SPR; cell aggregation; cell surface binding	(Lin et al. 2018; Choi et al. 2016; Li et al. 2015; Mah et al. 2010)
Slitrk1–6	Ig2–3	LRR	meB	Crystal structures (4RCA, 4Y61); SPR; cell aggregation; cell surface binding	(Yamagata, Sato, et al. 2015; Um et al. 2014; Yim et al. 2013)
NLGN3	Ig1–3	Cholinesterase‐like	meB exclusion	Crystal structures (7CEG); cell aggregation; cell surface binding; synaptogenesis assay	(Yoshida et al. 2021)

*Note:* This table catalogs postsynaptic partners of presynaptic PTPRD, indicating (i) the PTPRD domain(s) mediating binding, (ii) the corresponding partner domain(s), (iii) mini‐exon (meA/meB) dependence inferred from structural/biophysical and cell‐based assays, and (iv) the highest‐tier evidence supporting each interaction (e.g., crystal structure with PDB ID, SPR, cell‐aggregation/surface‐binding, synaptogenesis assays).

Abbreviations: FNIII, fibronectin type III domain; Ig, immunoglobulin‐like domain; IL1RAP, interleukin‐1 receptor accessory protein; IL1RAPL1, interleukin‐1 receptor accessory protein‐like 1; LRFN4/5 (SALM3/5), leucine‐rich repeat and FNIII domain–containing proteins 4/5 (synaptic adhesion‐like molecule 3/5); LRR, leucine‐rich repeat; LRRC4B (NGL‐3), leucine‐rich repeat–containing 4B (netrin‐G ligand‐3); meA/meB, PTPRD microexons A/B; NLGN3, neuroligin‐3; PDB, Protein Data Bank (e.g., 4YH7, 4YFD, 5XNP, 4RCA, 4Y61, 7CEG); PTPRD, protein tyrosine phosphatase receptor type D; Slitrk1–6, SLIT and NTRK‐like family proteins 1–6; SPR, surface plasmon resonance.

The Ig‐like domains also include two alternatively spliced mini‐exons, termed mini‐exon A (meA) and mini‐exon B (meB), which encode 9 and 4 amino acid residues, respectively (Figure 1B). The meA can be further subdivided into meA3 and meA6, and together with meB, these splice inserts critically regulate the trans‐synaptic binding specificity of PTPRD (Figure 1C). Inclusion of meA enables PTPRD to bind selectively to IL1RAPL1 (Yoshida et al. 2011; Yamagata, Yoshida, et al. 2015), a postsynaptic adhesion molecule regulating excitatory synapses and dendritic spines (Pavlowsky et al. 2010; Montani et al. 2019; Valnegri et al. 2011; Yoshida et al. 2011; Yasumura et al. 2014; Hayashi et al. 2013; Ramos‐Brossier et al. 2015). The meB is essential for interactions with IL1RAP (Yoshida et al. 2012), Slitrks (Yim et al. 2013; Takahashi et al. 2012), and LRFN4/5 (SALM3/5) (Lie et al. 2018, 2015; Mah et al. 2010; Lin et al. 2018; Goto‐Ito et al. 2018), whereas exclusion of meB facilitates binding to neuroligin‐3 (NLGN3) (Yoshida et al. 2021). Notably, earlier cell‐aggregation assays indicated that meB suppresses the PTPRD–LRFN5/SALM5 interaction (Choi et al. 2016), whereas later structural and surface plasmon resonance work observed robust PTPRD–SALM5 binding (including SALM5‐induced PTPRD dimerization) using constructs with or without meB, and did not replicate strong meB‐dependent suppression (Lin et al. 2018; Goto‐Ito et al. 2018).

Notably, neither meA nor meB affects the interaction between PTPRD and LRRC4B (NGL‐3), which involves the FNIII domains rather than the Ig‐like domains of PTPRD (Kwon et al. 2010; Woo, Kwon, Choi, et al. 2009; Woo, Kwon, Kim, et al. 2009). Structural insights into these trans‐synaptic adhesion complexes have been provided by crystallographic studies (Yamagata, Sato, et al. 2015; Won et al. 2019; Yamagata, Yoshida, et al. 2015; Lin et al. 2018; Goto‐Ito et al. 2018).

The meA3 and meA6 variants of the first mini‐exon (meA) encode distinct short loops that modulate PTPRD ligand recognition. Structural data show that the meA3 insert preserves the Ig2 fold but, on its own, does not provide the interactions needed for high‐affinity binding to postsynaptic partners such as IL1RAPL1 (Yamagata, Yoshida, et al. 2015). By contrast, isoforms containing meA6 (together with the meB linker) or the longer meA9 loop establish direct contacts between PTPRD and the Ig1 domain of IL1RAPL1, enabling the formation of a high‐affinity trans‐synaptic complex. Beyond meA and meB, two additional mini‐exons, meC embedded in the fifth FNIII domain and meD positioned immediately before the D1 domain, have been identified in LAR‐RPTPs (Won and Kim 2018), but their functional consequences remain unknown (Cornejo et al. 2021).

PTPRD is synthesized as a ~220‐kDa pro‐protein that, like all LAR‐RPTPs, is constitutively processed in the Golgi by a furin‐like convertase at a conserved penta‐arginine motif. This S1 cleavage yields a non‐covalent heterodimer consisting of a 150‐kDa extracellular E‐subunit (Ig + FNIII repeats) and an 85‐kDa membrane P‐subunit containing the stalk, transmembrane helix, and tandem phosphatase domains (Streuli et al. 1992; Serra‐Pages et al. 1994; Meehan et al. 2012).

In other receptor‐type PTPs of the same family, the E‐subunit can be shed from the surface by ADAM10‐mediated α‐secretase activity (S2 site), and the remaining membrane stub undergoes γ‐secretase intramembrane proteolysis (S3 site) that liberates a soluble intracellular fragment (ICD). The ICD of PTPRK and PTPRF translocates to the nucleus and attenuates β‐catenin‐dependent transcription (Anders et al. 2006; Haapasalo et al. 2007). Whether PTPRD undergoes S2/S3 cleavages remains to be determined.

The cytoplasmic region of PTPRD contains two tandem phosphatase homology domains. The membrane‐proximal domain (D1) harbors an intact protein tyrosine phosphatase (PTP) signature motif and is catalytically active. It has been shown to dephosphorylate several neuronal proteins, including TrkB and STAT3 (Tomita et al. 2020; Veeriah et al. 2009), and is hypothesized to regulate presynaptic protein turnover and neurotransmitter release probability, although this remains to be directly demonstrated in synaptic contexts.

D2 lacks the catalytic cysteine and behaves as a pseudo‐phosphatase scaffold. Crystal structures reveal that D2 binds the tandem SAM cassette of liprin‐α proteins through a conserved two‐site interface (Wakita et al. 2020). Via liprin‐α, which is itself a master organizer of the active zone (Xie et al. 2021; Spangler and Hoogenraad 2007), PTPRD is thought to regulate presynaptic proteins such as RIM1 and CASK (Spangler et al. 2013; Jin and Garner 2008; Sudhof 2012). Whether RIM1 and CASK are directly dephosphorylated by PTPRD remains unclear.

## 3 Spatiotemporal PTPRD Expression

Transcriptomic profiling (Human Protein Atlas, v23.1) classifies PTPRD as tissue‐enhanced, with the highest RNA levels in brain and parathyroid samples and lower but measurable levels in testis, adrenal and a few other tissues; immunohistochemistry confirms moderate cytoplasmic staining in selected non‐neural epithelia (Uhlen et al. 2015).

In the mouse brain, in situ hybridization and RNAscope revealed broad yet regionally accentuated mRNA expression: strong signals in hippocampal CA2/CA3, the thalamic reticular nucleus, cortical layer I, olfactory bulb, cerebellum, and spinal motor neurons, with lighter labeling elsewhere (Schaapveld et al. 1998; Kwon et al. 2010; Mizuno et al. 1993; Sommer et al. 1997; Shishikura et al. 2016). A PTPRD‐specific monoclonal antibody revealed PTPRD protein expression in brain regions, including olfactory bulb, cortex, hippocampus, cerebellum, and brain stem (Shishikura et al. 2016). An endogenous *Ptprd‐tdTomato* knock‐in mouse corroborates this distribution but also highlights axon‐rich laminae (layer I, stratum lacunosum‐moleculare, corpus callosum, and anterior commissure) as expression hotspots (Park et al. 2020).

Single‐nucleus RNA‐seq from the Allen whole‐brain cell‐type atlas shows robust *Ptprd* transcription in intra‐telencephalic and extra‐telencephalic excitatory neurons as well as multiple GABAergic subtypes, moderate expression in oligodendrocytes and oligodendrocyte precursors, and low expression in astrocytes and microglia (Yao et al. 2023). PTPRD‐tdTomato fluorescence is confined to tau‐positive axons and excluded from MAP2‐positive dendrites; ultrastructural quantification places ~80% of immunogold particles in vGluT2‐positive excitatory boutons, ~10% in nonsynaptic axon shafts and < 10% in GAD‐positive inhibitory terminals (Park et al. 2020) (Figure 1D).

Along the developmental timeline, *Ptprd* mRNAs are already detectable at embryonic Day 11 and rise steadily through late gestation (Sommer et al. 1997; Schaapveld et al. 1998). During this neurogenic window, PTPRD in Pax6^+^ radial glia and Tbr2^+^ intermediate progenitors limits TrkB/PDGFRβ→MEK–ERK signaling and restrains neuronal overproduction (Tomita et al. 2020). As corticogenesis shifts to gliogenesis (~E16–P0), *Ptprd* expression peaks; conditional ablation in telencephalic precursors (*Emx1‐Cre*) diminishes Sox9^+^ radial glia, blunts JAK/STAT activation and reduces astro‐ and oligodendro‐genesis (Cornejo et al. 2024). At E18.5, notably, *Ptprd* mRNAs are also detectable in the ventral gray matter motor neurons of the spinal cord (Zhu et al. 2015). Postnatally, PTPRD is transiently up‐regulated in maturing oligodendrocytes (P3–P15). *Ptprd* ^−/−^ mice display a brief delay in compact‐myelin formation that resolves by adulthood, indicating a permissive rather than obligatory role in myelination (Zhu et al. 2015).

In juvenile and adult mice, PTPRD‐tdTomato signals remain high, particularly along long‐range excitatory tracts (corpus callosum, anterior commissure) and layer‐I/molecular layers, consistent with the view that PTPRD persists as a presynaptic organizer throughout life, with region‐specific enrichment along axonal projection pathways (Park et al. 2020). Supporting this presynaptic role, PTPRD‐tdTomato fluorescence is confined to tau‐positive axons (Park et al. 2020). Functionally, presynaptic LAR‐RPTPs (including PTPRD) are required to sustain postsynaptic NMDAR content at SC‐CA1 synapses. In support of this notion, CA3‐restricted triple conditional knockout (cKO; PTPRD/PTPRS/PTPRF) selectively reduces NMDAR‐EPSCs and the NMDAR/AMPAR ratio with preserved synapse density and AMPAR transmission, and presynaptic Ptprs deletion recapitulates this NMDAR‐specific deficit (Sclip and Sudhof 2020; Kim et al. 2020; Horn et al. 2012). Moreover, cis associations of LAR‐RPTPs with neurexins suggest presynaptic nanodomain complexes that couple adhesion, release machinery, and phosphotyrosine signaling (Han et al. 2020). Nonetheless, postsynaptic contributions likely exist in some contexts; that is, RNAi or dominant‐negative LAR‐RPTP perturbations reduce dendritic spine density and AMPAR currents in cultured neurons (Dunah et al. 2005). We thus avoid over generalization and weigh ultrastructural and pathway‐specific evidence heavily.

Together, these results suggest that PTPRD is a brain‐enriched, neuron‐centric phosphatase whose expression is spatially, cellularly, and temporally tuned to the tasks it performs. It is transcribed at moderate levels in a few peripheral organs but reaches its highest abundance in the CNS, where mRNA and protein concentrate in axon‐rich laminae and in projection neurons of the hippocampus, cortex, olfactory bulb, thalamic reticular nucleus, cerebellum, and spinal cord. Single‐cell atlases confirm that expression is almost exclusively neuronal (both glutamatergic and GABAergic) while glia contribute little. Developmentally, PTPRD is detectable by embryonic Day 11, peaks around the neurogenesis–gliogenesis transition, and then remains robust in axons throughout life. These dynamics align with function: early PTPRD restrains progenitor proliferation and later supports gliogenesis; in the early postnatal period it accelerates initial myelination; and in juvenile–adult stages it serves as a presynaptic scaffold that is especially enriched along long‐range excitatory tracts. Together, the expression profile underscores PTPRD's dual identity as a developmental brake on cortical cell‐fate programs and a lifelong architect of synaptic connectivity.

## 4 PTPRD Mutations in Brain Disorders

A growing genetic literature places PTPRD among a small set of synaptic genes with pleiotropic neuropsychiatric impact (Table 2). The most consistent associations are for restless legs syndrome (Kim et al. 2013; Moore et al. 2014; Schormair et al. 2008; Yang et al. 2011; Didriksen et al. 2020; Schormair et al. 2017). Additional signals have been reported for addiction (Drgonova et al. 2015; Uhl et al. 2018; Drgon et al. 2010; Uhl and Martinez 2019) and for Alzheimer's disease‐related tau pathology (neurofibrillary tangle burden) (Chibnik et al. 2018). Gene‐ or SNP‐level genome‐wide signals have also been reported for insomnia (Jansen et al. 2019; Watanabe et al. 2022), obsessive compulsive disorder (OCD) (Burton et al. 2021; Mattheisen et al. 2015), and anxiety (Li et al. 2024), whereas ADHD, bipolar disorder, and anorexia nervosa show recurrent rare CNVs and suggestive common‐variant hits (Anney et al. 2008; Distel et al. 2011; Elia et al. 2010; Jarick et al. 2014; Malhotra et al. 2011; Walker et al. 2025). Rare loss‐of‐function or micro‐deletion alleles underlie intellectual disability with craniofacial anomalies (Choucair et al. 2015) and appear in multiplex cohorts of autism spectrum disorders (ASD) (Yang et al. 2024). Taken together, the strength of association varies widely, from genome‐wide significance (RLS, Alzheimer's disease) to early‐stage or provisional links. Continuous re‐evaluation of new large‐scale sequencing and GWAS datasets will be essential to refine PTPRD's disease spectrum.

**TABLE 2:** Human disease associations for PTPRD.

Disease/Trait	Human genetic evidence (study type)	Representative association strength	Animal model support	References
Restless legs syndrome (RLS)	GWAS/Meta‐analysis (EUR); replicated across independent cohorts	Genome‐wide significant locus; modest common‐variant effect	*Ptprd* ^−/−^ mice show altered locomotion and reduced non‐REM sleep; splice‐dependent sleep phenotypes (*Ptprd‐meA* mutants; *Emx1‐Cre*; *Ptprd* ^*fl/fl*^)	(Kim et al. 2013; Moore et al. 2014; Schormair et al. 2008; Yang et al. 2011; Park et al. 2020; Didriksen et al. 2020; Schormair et al. 2017)
Addiction (cocaine reward)	GWAS‐style pooled/candidate association studies in substance‐use cohorts	Suggestive associations; cohort‐dependent; not consistently genome‐wide significant	*Ptprd* loss or pharmacological antagonism reduces cocaine CPP/reward	(Drgonova et al. 2015; Uhl et al. 2018; Drgon et al. 2010; Uhl and Martinez 2019)
Alzheimer's disease/neurofibrillary tangle burden	Autopsy‐based GWAS of NFT (tau) pathology; replicated	Genome‐wide significant locus for NFT burden (endophenotype)	Aged *Ptprd* ^−*/−*^ mice show tau hyperphosphorylation and impaired cognition	(Chibnik et al. 2018; Foncea et al. 2025)
Insomnia/Sleep traits	GWAS/meta‐analysis; gene‐based test (MAGMA)	Genome‐wide significant gene in meta‐analysis	*Ptprd* ^−/−^ and cortical conditional mutants show reduced non‐REM sleep; splice‐dependent effects	(Jansen et al. 2019; Park et al. 2020; Watanabe et al. 2022)
Obsessive‐compulsive disorder/pediatric OC traits	GWAS (pediatric OC traits); GWAS (adult OCD)	Genome‐wide significant (pediatric OC traits); suggestive in adult OCD (cohort‐dependent)	*Ptprd* ^−/−^ mice show deficits in nest building and female‐specific PPI impairments	(Burton et al. 2021; Mattheisen et al. 2015; Ho et al. 2023)
Anxiety disorders	GWAS meta‐analysis with functional genomics (multi‐ancestry)	Genome‐wide significant locus including the PTPRD region	Anxiety‐like phenotypes variably reported; hyperactivity/mixed anxiety behaviors in some *Ptprd* mutants	(Li et al. 2024; Park et al. 2020)
Attention‐deficit/hyperactivity disorder (ADHD)	Rare CNV studies (case–control; de novo trios)	Recurrent, cohort‐dependent CNVs; no genome‐wide significant common‐variant association	Hyperactivity in *Ptprd* ^−/−^ mice	(Elia et al. 2010; Jarick et al. 2014; Park et al. 2020)
Schizophrenia	CNV burden meta‐analyses and case–control studies	Global CNV burden enriched; PTPRD gene‐level CNV signals are sporadic/cohort‐dependent; not a consistent common‐variant GWAS locus	Unclear. No schizophrenia‐specific model	(Malhotra et al. 2011; Cortes et al. 2024; Marshall et al. 2017; International Schizophrenia 2008; Kirov et al. 2012)
Bipolar disorder	De novo CNV studies (trios/case‐parent)	Increased de novo CNV burden reported in some cohorts (includes PTPRD duplication); replication mixed across studies	Unclear. No bipolar‐specific phenotypes established	(Malhotra et al. 2011; Georgieva et al. 2014)
Anorexia nervosa	Genome‐wide CNV association (case–control; ANGI)	PTPRD CNV region shows nominal association (not genome‐wide significant); replication pending	Indirect. Strong metabolic phenotypes via asprosin–PTPRD axis but not an anorexia model	(Walker et al. 2025; Mishra et al. 2022)
Autism spectrum disorder	WES/WGS rare coding‐variant & CNV studies (de novo + inherited); hybrid trio+population common‐variant analysis	Moderate rare‐variant support in subsets; suggestive common‐variant signal (FDR‐controlled; below classical genome‐wide significance)	Ptprd^−/−^ mice show social‐interaction deficits and repetitive grooming; splice‐mutant mice show repetitive rearing	(Yang et al. 2024; Cortes et al. 2024; Kim et al. 2025; Toma et al. 2013; Yuen et al. 2017; Zhou et al. 2022)
Intellectual disability (with craniofacial anomalies)	Case report—homozygous microdeletion affecting PTPRD (chromosomal microarray)	Pathogenic microdeletion (qualitative; single family)	Partial. Broad neurodevelopmental phenotypes in *Ptprd* mutants (migration, dendrite, synapse)	(Choucair et al. 2015; Tomita et al. 2020; Nakamura et al. 2017)
Metabolic syndrome/obesity‐related traits	GWAS/meta‐analyses: no consistent PTPRD association; pharmacogenetic candidate studies in treatment‐induced weight gain	No replicated genome‐wide significant association; cohort‐specific pharmacogenetic findings require replication	Global or AgRP‐specific *Ptprd* deletion abolishes asprosin responses; anti‐asprosin mAbs or soluble PTPRD decoy reduce intake and weight	(Mishra et al. 2022; Mishra et al. 2021)

*Note:* This table summarizes human genetic evidence linking PTPRD to disorders/traits alongside representative effect strength and convergent support from mouse models.

Abbreviations: ADHD, attention‐deficit/hyperactivity disorder; AgRP, agouti‐related peptide; ANGI, Anorexia Nervosa Genetics Initiative; ASD, autism spectrum disorder; CNV, copy‐number variant; CNVR, copy‐number variant region; CPP, conditioned place preference; DIO, diet‐induced obesity; EAS, East Asian ancestry; Emx1‐Cre, telencephalon‐restricted Cre driver; EUR, European ancestry; FDR, false discovery rate; GWAS, genome‐wide association study; mAb/mAbs, monoclonal antibody/antibodies; MAGMA, Multi‐marker Analysis of GenoMic Annotation (gene‐based test); meA, PTPRD mini‐exon A; NFT, neurofibrillary tangle; non‐REM, non‐rapid eye movement sleep; OC, obsessive‐compulsive (traits); OCD, obsessive‐compulsive disorder; OR, odds ratio; PPI, prepulse inhibition; PTPRD, protein tyrosine phosphatase receptor type D; *Ptprd* ^−/−^, global Ptprd knockout; *Ptprd* ^*fl/fl*^, homozygous floxed Ptprd allele; RLS, restless legs syndrome; SUD, substance use disorder; WES, whole‐exome sequencing; WGS, whole‐genome sequencing.

## 5 Behavioral Abnormalities in *Ptprd*‐Mutant Mice

Mutant‐mouse work now spans many behavioral domains linked to human PTPRD genetics. Global *Ptprd*‐KO (*Ptprd* ^−/−^) mice display enhanced CA1/CA3 LTP with decreased spatial learning (Uetani et al. 2000), establishing a role in learning and memory. *Ptprd* ^−/−^ mice show sleep dysfunctions, supported by suppressed sleep behavior and non‐REM sleep (Park et al. 2020; Drgonova et al. 2015), consistent with the association with restless legs syndrome. Suppressed non‐REM sleep is also observed in *Ptprd‐meA*‐KO (*Ptprd‐meA* ^−/−^) mice (mini‐exon A‐mutant; see below) and cortical, conditional *Ptprd*‐KO mice (*Emx1‐Cre*; *Ptprd* ^fl/fl^) (Park et al. 2020), adding circuit and cell‐type contexts.

Locomotive and anxiety‐related abnormalities are also observed. Hyperactivity and mixed anxiety‐like behaviors were observed in *Ptprd* ^−/−^ mice (Park et al. 2020). Heterozygous *Ptprd‐meA* ^+/−^ mice showed normal locomotion and anxiety‐like behaviors, whereas *Ptprd‐meB* ^+/−^ mice exhibited hypoactivity and anxiety‐like behaviors (Kim et al. 2025). Notably, *Ptprd‐meB* ^−/−^ mice show strongly increased (~3‐fold) early postnatal lethality (Kim et al. 2025), in line with the developmental functions of PTPRD.

Addiction, OCD, and autism‐related behaviors have been reported. *Ptprd* ^−/−^ mice show reduced cocaine reward (Drgonova et al. 2015; Uhl et al. 2018) and deficits in nest building (a goal‐directed behavior) and prepulse inhibition (only in females) (Ho et al. 2023). *Ptprd* ^−/−^ and *Ptprd* ^+/−^ mice show autistic‐like behaviors, including social deficits (social interaction and social novelty recognition) and repetitive behaviors (self‐grooming and marble burying), while learning/memory and anxiety measures were spared despite testing with Y‐maze, Morris water maze, and elevated plus maze (Cortes et al. 2024). *Ptprd‐meB* ^+/−^ mice also show ASD‐like repetitive rearing in the Laboras test, a familiar environment (Kim et al. 2025).

Aged *Ptprd* ^−/−^ mice display tau hyperphosphorylation, impaired spatial learning and memory, and proinflammatory microgliosis (Foncea et al. 2025). The *Ptprd* ^−/−^ cerebellum shows impaired climbing‐fiber synapse development and poor rotarod performance (Okuno et al. 2023), while a sextuple Purkinje‐cell deletion of neurexins plus PTPRD/PTPRS/PTPRF decreases Purkinje cell synapses on deep cerebellar nuclei and precipitates motor dysfunctions (Sclip and Sudhof 2023).

Lastly, extra‐synaptic functions translate into metabolic and motor outputs: AgRP‐neuron or body‐wide deletion of PTPRD abolishes asprosin signaling, causing hypophagia and resistance to diet‐induced obesity (Mishra et al. 2022). In addition, cerebellar Purkinje cell‐specific deletion of PTPRD (*L7‐Cre*; *Ptprd* ^fl/fl^) suppresses baseline and asprosin‐induced water intake (Mishra et al. 2024), suggesting that the asprosin‐PTPRD axis regulates thirst.

These findings (summarized in Table 3) indicate that *Ptprd* deletion in mice leads to a spectrum of behavioral abnormalities. It should be noted, however, that constitutive KO phenotypes, while potentially reflecting disease‐relevant whole‐brain conditions, may also arise from pleiotropic effects across both neuronal and non‐neuronal lineages. More direct insights into specific PTPRD functions can therefore be achieved using localized or cell type–restricted manipulations, such as conditional KOs, projection‐specific deletions, or stereotactic AAV‐mediated approaches. It should also be noted that certain behavioral phenotypes differ even among mouse lines that share the same constitutive *Ptprd* deletion. For example, Uetani et al. (2000) reported that global *Ptprd* ^−/−^ mice exhibited impaired spatial learning in the Morris water maze, while Park et al. (2020) found that *Ptprd* ^−/−^ mice displayed hyperactivity and mixed anxiety‐like behaviors. In contrast, Cortes et al. (2024) observed that *Ptprd* ^−/−^ mice did not exhibit learning or memory impairments, nor anxiety‐related abnormalities. Several factors likely contribute to these apparent discrepancies, including differences in genetic background or substrain, behavioral batteries and task parameters, age and sex composition, housing and handling conditions, and allele design, which can influence developmental compensation among LAR‐RPTPs. Consequently, cognitive and anxiety‐related outcomes appear to be highly context‐dependent.

**TABLE 3:** Region/Model comparison of synaptic and behavioral phenotypes for PTPRD mutations in mice.

Brain region/circuit	Mouse model	Synaptic/Neuronal function and splice dependence	Synaptic phenotype	Behavioral/Physiological phenotype	Mouse age	References
Hippocampus; SC → CA1, CA3	Global *Ptprd* ^−/−^ (constitutive KO)	Synaptic plasticity; Presynaptic	LTP ↑ at SC → CA1/CA3 PPF ↑ at CA1/CA3 (release probability ↓)	Growth retardation Semi‐lethality Impaired spatial learning	Juvenile–adult	(Uetani et al. 2000)
Whole brain	Global *Ptprd* ^+/−^ and *Ptprd* ^−/−^			Body weight ↓ at *Ptprd* ^+/−^ and *Ptprd* ^−/−^ Strength/motor persistence ↓ at *Ptprd* ^−/−^ Hyperactivity (Cocaine‐conditioned place preference) at *Ptprd* ^−/−^ Sleep disturbance at *Ptprd* ^−/−^ Impaired spatial learning at *Ptprd* ^−/−^ Cocaine reward ↓ at *Ptprd* ^−/−^	Adult	(Drgonova et al. 2015)
Hippocampus; SC → CA1	Global TKO (*Ptprd/s/f*) and CA3‐restricted cTKO (*Ptprd/s/f*)	Trans‐synaptic NMDAR regulation	mEPSC freq ↓ at Global TKO NMDAR‐eEPSC amp ↓ (AMPAR‐eEPSC amp unchanged) at Global TKO NMDAR/AMPAR ratio ↓ at Global TKO and CA3‐cTKO		Juvenile	(Sclip and Sudhof 2020)
Hippocampus; EC → CA1 (SLM layer)	Global *Ptprd* ^−/−^, *Ptprd‐meA* ^−/−^, and *Emx1*; *Ptprd* cKO	Synapse development; meA‐dependent PTPRD—IL1RAPL1	Input/output ratio ↓ at *Ptprd* ^−/−^ and *Ptprd‐meA* ^−/−^	PSD density ↓ at *Ptprd* ^−/−^ Presynaptic terminal density ↓ at *Ptprd* ^−/−^ Hyperactivity (Open‐field test/OFT) at *Ptprd* ^−/−^, *Ptprd‐meA* ^−/−^ and *Emx1*; *Ptprd* cKO Sleep disturbance at *Ptprd* ^−/−^, *Ptprd‐meA* ^−/−^ and *Emx1*; *Ptprd* cKO Non‐REM sleep & delta‐power ↓ at *Ptprd‐meA* ^−/−^ and *Emx1*; *Ptprd* cKO	Juvenile‐adult	(Park et al. 2020)
Hypothalamus; AgRP neurons	Global *Ptprd* ^−/−^, *Ptprd* ^+/−^, and *AgRP*; *Ptprd* cKO (+PTPRD inhibitor)	Receptor–ligand signaling (asprosin → PTPRD)	Impaired asprosin‐induced firing rate and depolarization at *Ptprd* ^−/−^ Impaired SK current amp, firing rate and RMP at *AgRP* ^*ARH*^ (+PTPRD inhibitor) & *AgRP*; *Ptprd* ^−/−^	Body weight and food intake (orexigenic responses) ↓ STAT3 dephosphorylation is PTPRD‐dependent and ↓ at *Ptprd* ^−/−^	Adult	(Mishra et al. 2022; Feng et al. 2023)
Cerebellum; CF → PC, PF → PC	Global *Ptprd* ^−/−^	Synapse development	Asynchronous EPSC freq ↓ at *Ptprd* ^−/−^ (CF → PC) CF‐EPSC amp ↓ PPR ↓ at *Ptprd* ^−/−^ (CF → PC) (release probability ↑) PF‐EPSC amp ↑	Body weight ↓ CF innervation ↓ CF synapse size and formation ↓ Motor dysfunction	Postnatal‐juvenile‐young adult	(Okuno et al. 2023)
Cerebellum; PC → DCN	PV;3cKO (*Nrxn1/2/3 or Ptprd/s/f*) PV; 6cKO (*Nrxn1/2/3 +Ptprd/s/f*)	Synapse development; Combined effects of LAR‐RPTPs & neurexins	mIPSC freq ↓ at PV; 6cKO eIPSC amp ↓ at PV;6cKO	Severe motor dysfunction Inhibitory synaptic density ↓ Inhibitory synaptic volume ↓ PC → DCN synapse ↓	Juvenile	(Sclip and Sudhof 2023)
Whole brain	Global *Ptprd* ^+/−^ and *Ptprd* ^−/−^			Goal‐directed behavior ↓ at *Ptprd* ^−/−^ PPI ↓ (Female) at *Ptprd* ^−/−^	Adult	(Ho et al. 2023)
Hippocampus; CA1	*Ptprd* cKO and *Ptprd‐meA* cKO; CA3 → dCA1 SuB → dCA1, EC → dCA1	Trans‐synaptic NMDAR regulation; meA‐dependent PTPRD—IL1RAPL1	NMDAR/AMPAR ratio ↑ at *Ptprd* cKO (CA3 → dCA1/SuB → dCA1) and *Ptprd‐meA* cKO (SuB → dCA1) Asynchronous EPSC amp ↓ at *Ptprd* cKO (SuB → dCA1) Asynchronous EPSC freq ↓ at *Ptprd* cKO (EC → dCA1)	Impaired object‐location memory at *Ptprd* cKO (CA3 → dCA1/SuB → dCA1) and *Ptprd‐meA* cKO (SuB → dCA1)	Adult	(Han et al. 2024)
Cerebellum; PC	Global *Ptprd* ^−/−^ and *L7*; *Ptprd* ^−/−^	Receptor–ligand signaling (asprosin→PTPRD)	Impaired water‐deprivation‐induced firing rate and RMP	Water intake ↓ Impaired asprosin‐induced thirst	Adult	(Mishra et al. 2024)
mPFC; layer 2/3	Global *Ptprd* ^+/−^ and *Ptprd* ^−/−^	Synapse development	m/sEPSC and m/sIPSC freq ↑ at *Ptprd* ^−/−^ Input/output ratio ↑ at *Ptprd* ^−/−^ eIPSC amp ↑ at *Ptprd* ^−/−^	Impaired social interaction and social novelty‐recognition at *Ptprd* ^+/−^ and *Ptprd* ^−/−^ Marble burying ↑ at *Ptprd* ^+/−^ and *Ptprd* ^−/−^ Self‐grooming ↑ at *Ptprd* ^−/−^	Adult	(Cortes et al. 2024)
Hippocampus; EC → DG (granule cells)	*Ptprd‐meB* ^+/−^, *Ptprd‐meA* ^+/−^, *Ptprd‐meB* ^+/fl^ (MEC AAV‐Cre; conditional heterozygote); MEC → DG, *Il1rap* ^+/−^	Synapse development; meB‐dependent PTPRD—IL1RAP	mEPSC freq ↓ at *Ptprd‐meB* ^+/−^ eEPSC/eIPSC ratio ↓ at *Ptprd‐meB* ^+/−^, *PtprdmeB cHT* (MEC → DG; MEC AAV‐Cre + *Ptprd‐meB* ^+/fl^), and *Il1rap* ^+/−^ oEPSC/oIPSC ratio ↓ at *Ptprd‐meB* ^+/−^ oEPSC/oIPSC ratio ↑ at *Ptprd‐meA* ^+/−^	Anxiety (OFT, Elevated plus‐maze/EPM & Light–dark/LD) ↑ at *Ptprd‐meB* ^+/−^ PSD density ↓ at *Ptprd‐meB* ^+/−^	Adult	(Kim et al. 2025)
Hippocampus; EC → DG (interneurons)	*Ptprd‐meB* ^+/−^	Synapse development	mEPSC freq ↑	Excitatory synaptic density ↑	Adult	(Kim et al. 2025)
Cortex and Hippocampus	Global *Ptprd* ^−/−^			Tau phosphorylation ↑ Abl kinase activation ↑ (Hippocampus) Impaired spatial learning Microgliosis ↑ PSD density ↓ (Cortex)	Aged adult	(Foncea et al. 2025)

*Note:* The table cross‐indexes brain region/circuit, genetic model (global or Cre‐restricted; single, triple, or sextuple deletions), splice dependence (meA/meB), and the principal synaptic/neuronal and behavioral outcomes reported in the cited studies.

Abbreviations: 3cKO/6cKO, triple/sextuple conditional knockout; AgRP, agouti‐related peptide; ARH, arcuate nucleus of the hypothalamus; CA1/CA3, hippocampal cornu ammonis fields 1/3; CF, climbing fiber; cKO, conditional knockout; cTKO, conditional triple knockout; dCA1, distal CA1; DCN, deep cerebellar nuclei; DG, dentate gyrus; EC, entorhinal cortex; eEPSC, evoked excitatory postsynaptic current; Emx1‐Cre, telencephalon‐restricted Cre driver; EPM, elevated plus‐maze; EPSC, excitatory postsynaptic current; IL1RAP/Il1rap, interleukin‐1 receptor accessory protein (protein/gene); IL1RAPL1, interleukin‐1 receptor accessory protein‐like 1; KO, knockout; L7, Purkinje cell protein 2; Pcp2; Purkinje cell–specific Cre driver; LAR‐RPTPs, type IIa receptor protein tyrosine phosphatases (PTPRF/LAR, PTPRD, PTPRS); LD, light–dark box; LTP, long‐term potentiation; meA/meB, PTPRD mini‐exons A/B; MEC, medial entorhinal cortex; mEPSC/mIPSC, miniature EPSC/IPSC; mPFC, medial prefrontal cortex; NMDAR/AMPAR, NMDA‐/AMPA‐type glutamate receptor; Non‐REM, non‐rapid eye movement/REM sleep; Nrxn, neurexin; oEPSC/oIPSC, optogenetically evoked EPSC/IPSC; OFT, open‐field test; PC, Purkinje cell; PF, parallel fiber; PPF, paired‐pulse facilitation; PPI, prepulse inhibition; PPR, paired‐pulse ratio; PSD, postsynaptic density; PTPRD, protein tyrosine phosphatase receptor type D; RMP, resting membrane potential; SC, Schaffer collateral; sEPSC/sIPSC, spontaneous EPSC/IPSC; SK current, small‐conductance Ca^2+^‐activated K^+^ current; SLM, stratum lacunosum‐moleculare; STAT3, signal transducer and activator of transcription‐3; SuB, subiculum; TKO, triple knockout.

## 6 PTPRD Regulates Synapse Density In Vivo

Trans‐synaptic adhesions have been implicated in the regulation of synapse development and function (Gomez et al. 2021; Sudhof 2021; Sudhof 2023; Kim et al. 2021). The trans‐synaptic interactions of PTPRD and its paralogues position LAR‐RPTPs as modulators rather than builders of synapses. In CA3‐restricted triple conditional KOs lacking PTPRD, PTPRS, and PTPRF, Schaffer collateral‐CA1 (SC‐CA1) synapses retain normal excitatory synapse density, active‐zone structure, and baseline AMPAR‐EPSCs (Sclip and Sudhof 2020). In addition, a pan‐neuronal Synapsin‐Cre deletion of the same three genes leaves excitatory and inhibitory synapse number, vesicle clustering/docking, and release probability unchanged in cultured hippocampal neurons and in CA1 pyramidal cells in vivo; the only ultrastructural change is a modest (~30%) widening of the synaptic cleft (Emperador‐Melero et al. 2021). Together these studies show that LAR‐RPTPs are dispensable for synapse assembly under the tested condition but fine‐tune cleft architecture and are presynaptically essential for sustaining postsynaptic NMDAR content and plasticity.

**FIGURE 2:**
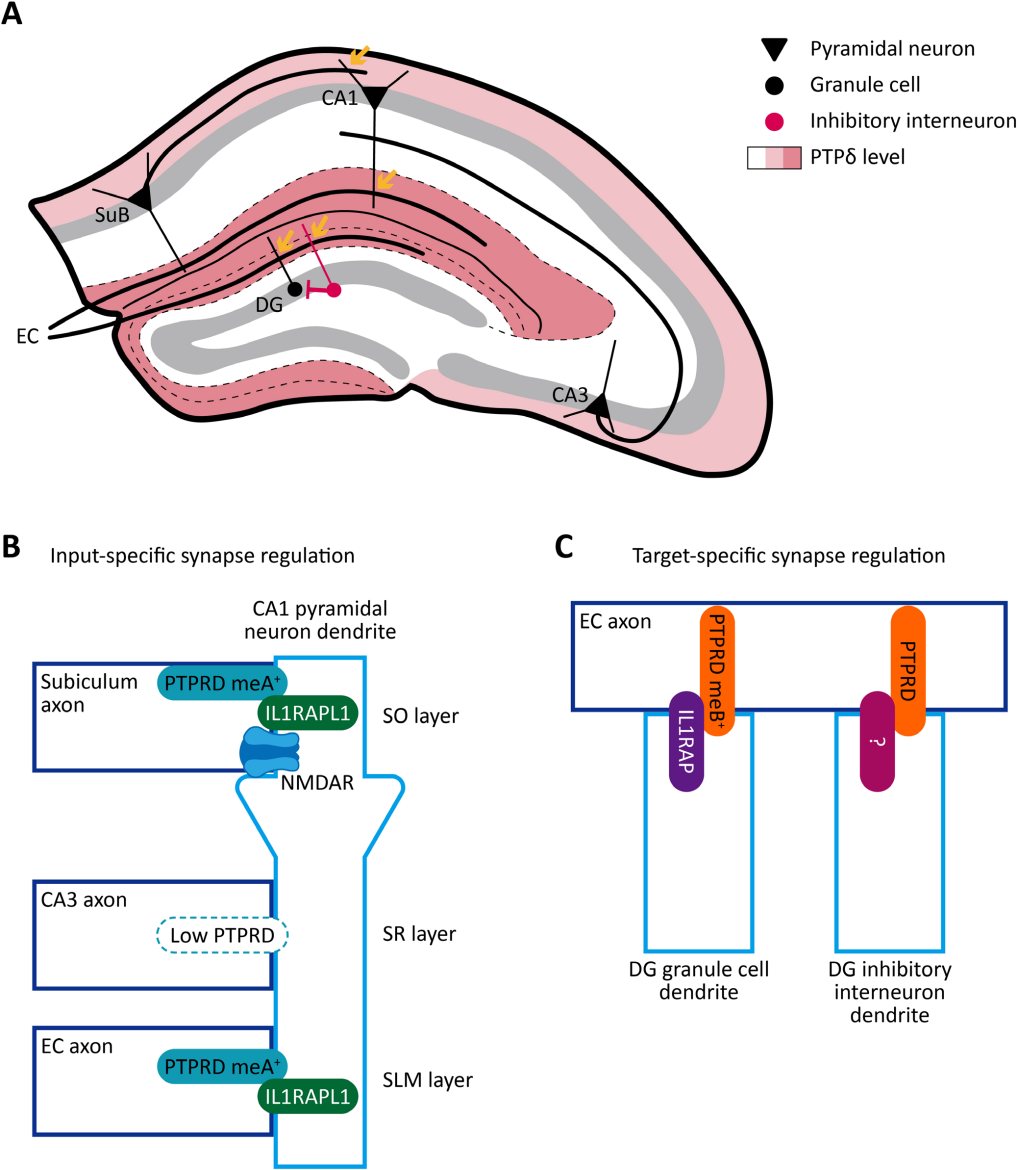
Target and input‐specific synapse regulation by PTPRD. (A) Schematic representation illustrating hippocampal regions, that is, the dentate gyrus (DG) and CA1, highlighting sites of target‐ and input‐specific synaptic regulation mediated by PTPRD. (B) Input‐specific synapse regulation by PTPRD. CA1 dendrites receive distinct excitatory synaptic inputs originating from entorhinal cortex (EC) axons in the SLM layer, CA3 axons in the SR layer, and subicular (SuB) axons in the SO layer. Notably, EC and SuB axons expressing PTPRD‐meA regulate CA1 synapses through trans‐synaptic PTPRD‐meA–IL1RAPL1 interactions, influencing synapse development and maintenance in the SLM layer, and modulating NMDAR responses in the SO layer. CA3 axons, which exhibit low PTPRD expression, show minimal functional changes upon its deletion in mice. (C) Target‐specific synapse regulation by PTPRD. EC axons projecting into the dentate gyrus (DG) region encounter two distinct postsynaptic neuron types: DG granule cells (DG‐GCs) and DG inhibitory interneurons (DG‐INs). Although the same EC axon terminals form excitatory synapses with both neuron types, only EC–DG‐GC synapses specifically require the PTPRD‐meB–IL1RAP interaction. In contrast, it remains unclear whether EC–DG‐IN synapses similarly depend on meB for their trans‐synaptic interactions.

Despite these network‐wide redundancies, several studies show that PTPRD is important for excitatory synapse density in specific pathways (Figure 2A,B). In global *Ptprd* ^−/−^ mice, the density of axon terminals apposed to the postsynaptic density (PSD) at entorhinal‐cortex to stratum‐lacunosum‐moleculare (EC–SLM) synapses falls by ~25%, as revealed by electron microscopy (Park et al. 2020). This vulnerability involves the PTPRD–IL1RAPL1 trans‐synaptic interaction, which requires the meA micro‐exon in PTPRD for high‐affinity binding (Yoshida et al. 2011; Yamagata, Yoshida, et al. 2015). By contrast, SC‐CA1 synapses in the proximal apical dendrites maintain normal evoked AMPAR transmission, consistent with preserved synapse number, both in the *Ptprd* ^−/−^ (field recordings), *Ptprd‐meA* ^−/−^ mice, and projection‐specific *Ptprd‐meA* ^−/−^ mice (Han et al. 2024). These results suggest input‐specific loss of excitatory synapses, indicating that PTPRD is essential in circuits that rely on its meA‐dependent adhesion code but dispensable where alternative organizers suffice.

A more recent study shows that the impact of PTPRD depends not only on the presynaptic splice code but also on the identity of the postsynaptic target cell (Figure 2C). In heterozygous *Ptprd‐meB* ^+/−^ mice (homozygotes are perinatally lethal) excitatory bouton density and AMPAR‐EPSCs from entorhinal‐cortex inputs onto dentate‐gyrus granule cells fall by ~30%–35%, even though PSD ultrastructure is unchanged (Kim et al. 2025). The deficit is reproduced in *Il1rap* ^−/−^ mice and therefore hinges on the meB‐dependent PTPRD–IL1RAP interaction (Yamagata, Yoshida, et al. 2015; Yoshida et al. 2012). Paradoxically, the same presynaptic deletion increases bouton density and EPSC amplitude onto neighboring molecular‐layer interneurons, demonstrating opposite, target‐specific outcomes from one splice variant.

In the cerebellum, simultaneous Purkinje‐cell deletion of all three LAR‐RPTPs (PTPRD/PTPRS/PTPRF) together with all three neurexins (Nrxn1/2/3) reduces the density of GABAergic Purkinje‐bouton contacts on deep‐cerebellar‐nucleus neurons and causes severe motor deficits (Sclip and Sudhof 2023). Either triple KO alone left bouton density intact, highlighting strong functional redundancy. Consistent with this genetic synergy, neurexins and LAR‐RPTPs can interact in cis to co‐organize presynaptic architecture (Han et al. 2020). These results suggest that combinatorial adhesion‐molecule networks are essential for building and maintaining functional cerebellar circuits, highlighting the need to view synapse organizers as cooperative rather than strictly nonredundant.

Collectively, these in vivo results (Table 3) indicate that PTPRD is a context‐dependent synapse organizer whose requirement varies by brain region, splice code, and target cell. In the hippocampus, PTPRD is dispensable for SC‐CA1 connectivity but required at cortical inputs: meA–IL1RAPL1 signaling supports EC–SLM synapses, while meB–IL1RAP signaling maintains EC–dentate granule inputs yet produces opposite effects on neighboring interneurons. In cortex, PTPRD loss can enhance connectivity, notably hyperconnectivity in mPFC, suggesting a role in balancing rather than building synapses. In the cerebellum, PTPRD partners with neurexins to organize Purkinje outputs and contributes to climbing fiber–Purkinje development, with redundancy masking single‐gene loss. Overall, PTPRD is essential where meA/meB adhesion codes or cooperative adhesion networks operate, but dispensable where alternative organizers suffice. Whether its loss disrupts initial contact, maturation, or both remains unresolved.

## 7 PTPRD Regulates Synaptic Function In Vivo

Multiple in vivo studies show that PTPRD shapes synaptic transmission and plasticity. In the earliest report, global *Ptprd* ^−/−^ mice displayed markedly enhanced LTP at SC‐CA1 synapses (and at CA3 recurrent synapses), while paired‐pulse facilitation rose only at CA1, suggesting a local decrease in presynaptic release probability (Uetani et al. 2000). These findings suggest two (not mutually exclusive) scenarios: (i) under normal conditions PTPRD helps keep release probability high, so its loss lowers release and thereby permits larger postsynaptic potentiation, or (ii) chronically elevated LTP caused by the KO triggers a homeostatic depression of presynaptic release.

In CA3‐specific triple cKO mice lacking *Ptprd*, *Ptprs*, and *Ptprf*, SC‐CA1 synapses show normal synapse density, active‐zone ultrastructure, and baseline AMPAR‐EPSCs, as mentioned above, yet NMDAR‐EPSCs and the NMDAR/AMPAR ratio drop by ~50% (Sclip and Sudhof 2020). Selective presynaptic deletion of PTPRS replicates this NMDAR‐specific deficit at SC‐CA1 synapses (Kim et al. 2020; Horn et al. 2012), suggesting a presynaptic requirement for LAR‐RPTPs in maintaining postsynaptic NMDAR content. Extending the theme of input specificity, projection‐restricted deletion of the meA‐containing PTPRD isoform from SC‐CA1 or Subiculum‐CA1 axons leaves synapse number, AMPAR‐EPSCs, and paired‐pulse facilitation intact, yet increases the NMDAR/AMPAR ratio and impairs object‐location memory (Han et al. 2024). These results collectively suggest that LAR‐RPTPs are dispensable for building synapses but are presynaptically essential for sustaining postsynaptic NMDAR function, and that alternative splicing of PTPRD further tunes this requirement on a pathway‐by‐pathway basis. However, an earlier study using RNA interference and dominant‐negative constructs targeting all three LAR‐RPTPs in cultured hippocampal neurons revealed reduced dendritic spine density and AMPAR‐mediated currents (Dunah et al. 2005), suggesting a potential postsynaptic and cell‐autonomous role of PTPRD in AMPAR regulation.

In the medial prefrontal cortex, loss of PTPRD reshapes cortical circuitry rather than simply weakening it. In constitutive KO mice, both excitatory and inhibitory neuron numbers remain elevated from development into adulthood, and layer 2/3 pyramidal cells show roughly two‐fold increases in the frequency of spontaneous EPSCs and IPSCs, while paired‐pulse ratios and AMPAR/NMDAR balance are unchanged (Cortes et al. 2024). These results indicate that PTPRD is essential for keeping excitatory and inhibitory synapse production in check; its absence produces hyper‐connected networks that translate into autism‐relevant behaviors.

In the cerebellum, *Ptprd* ^−/−^ mice display multiple defects in climbing‐fiber (CF) development (Okuno et al. 2023). Purkinje cells receive fewer CF inputs as early as P3–P7, and pruning of the remaining secondary fibers accelerates after P8. In anterior lobules the dominant CF fails to translocate fully onto distal dendrites (P12–P30) and CF‐evoked EPSCs stay 30%–50% smaller, whereas posterior lobules recover by P19. Paired‐pulse depression is enhanced, consistent with higher release probability, but whether the reduced EPSCs mainly reflect fewer synapses, altered postsynaptic receptor content, or both remains to be determined.

Overall, current evidence (Table 3) indicates that PTPRD is not required for the initial formation of synapses, but it plays a critical role in fine‐tuning synaptic properties, including release probability, NMDAR maintenance, and long‐term plasticity. Its functions are highly context‐dependent, shaped by brain region, splice isoform usage, and developmental stage. In many circuits, redundancy with other LAR‐RPTPs or neurexins likely masks the full impact of PTPRD loss, complicating the interpretation of KO phenotypes. Looking ahead, substrate‐trap mutants will be valuable for identifying direct presynaptic dephosphorylation targets. In addition, conditional manipulations in adulthood will help distinguish developmental roles from acute requirements, while cross‐regional comparisons across the hippocampus, prefrontal cortex, and cerebellum may clarify whether PTPRD primarily restrains, sustains, or enhances connectivity in a circuit‐specific manner.

## 8 PTPRD Regulates Protein Tyrosine Phosphorylation

PTPRD is a bifunctional receptor: its ectodomain mediates cell adhesion, whereas its cytoplasmic tail contains two phosphatase‐homology modules. The membrane‐proximal D1 domain is catalytically active; the distal D2 domain is enzymatically silent but binds liprin‐α scaffolds (Serra‐Pages et al. 1998). Liprin‐α family proteins are known to function as presynaptic scaffolds that drive active‐zone assembly across species (Zhen and Jin 1999; Spangler et al. 2013; Hoogenraad et al. 2007; Spangler and Hoogenraad 2007; Astigarraga et al. 2010) through their direct interaction with the D2 domain of LAR‐RPTPs (Xie et al. 2020; Wakita et al. 2020).

This arrangement raises two key questions: (i) does receptor clustering, driven by trans‐synaptic ligands or by lateral liprin‐α interactions, modulate D1 activity, and (ii) which presynaptic proteins are dephosphorylated by PTPRD and why does that matter? Evidence to date suggests that clustering inhibits the phosphatase. An antibody that forces PTPRD dimerization blocks D1 activity and triggers receptor degradation (Qian et al. 2023; Tremblay 2023); chemically induced D1 dimerization has the same effect. Further, the D2 domain of PTPRD can bind and inhibit the D1 domain of the paralogue PTPRS when the two are brought into close apposition (Wallace et al. 1998), and, in PTPRF, D2‐mediated clustering via liprin‐α suppresses D1 catalysis (Xie et al. 2020). Taken together, these results imply that trans‐synaptic or liprin‐α‐driven clustering of PTPRD would elevate pTyr levels on selected presynaptic proteins, altering their interactions, assembly, and function. Consistent with this idea, presynaptic induction by PTPRS requires liprin‐α binding but not phosphatase activity (Bomkamp et al. 2019; Han et al. 2018), suggesting that LAR‐type phosphatases shape mature synaptic signaling rather than the initial formation of synapses. Exactly which presynaptic targets are regulated by PTPRD, and how their dephosphorylation affects neurotransmission, remains a central open problem.

Pinpointing the physiological substrates of PTPRD at synapses remains a major gap. Outside the nervous system, biochemical and cell‐based studies show that PTPRD dephosphorylates STAT3 (Veeriah et al. 2009; Ortiz et al. 2014; Peyser et al. 2015; Yu et al. 2017) and AURKA, the latter leading to oncoprotein destabilization in neuroblastoma cells (Meehan et al. 2012). Within the developing brain, substrate trapping and phospho‐assays identify TrkB and PDGFRβ as PTPRD substrates in neural precursors, dampening MEK–ERK signaling and restricting the pool of intermediate progenitor cells (Tomita et al. 2020). In the embryonic cortex, PTPRD associates with the Sema3A/Neuropilin‐1 complex and activates Fyn by dephosphorylating its C‐terminal inhibitory tyrosine (Fyn Y531/Src Y527), thereby promoting dendritic growth; loss or catalytic inactivation of PTPRD keeps Fyn inhibited, and rescue with active PTPRD restores dendritic complexity (Nakamura et al. 2017). These examples (summarized in Table 4) suggest that PTPRD can act on receptor tyrosine kinases and signaling enzymes, but a comprehensive synaptic substrate map and an understanding of how each dephosphorylation event shapes neurotransmission remain to be established.

**TABLE 4:** Candidate substrates of PTPRD phosphatase activity.

Protein (common)	Gene (mouse)	UniProt (mouse)	Residue(s) affected	Evidence type	Primary context/note	Replicated across studies?	Representative reference
Fyn (Src family kinase)	Fyn	P39688	Y531 (C‐terminal inhibitory site)	Direct site mapping (Fyn Y531); biochemical + genetic evidence in the Sema3A–Nrp1–PTPRD axis; dendritic phenotype rescued by catalytically active (but not inactive) PTPRD	Embryonic cortex; PTPRD activation of Fyn promotes dendritic arborization	No (single primary study)	(Nakamura et al. 2017)
STAT3	Stat3	P42227	pY705 (activation site)	Direct dephosphorylation at Y705 by PTPRD, with supportive pathway data in later studies	Mouse hypothalamus AgRP neurons; Asprosin–PTPRD axis reduces STAT3 phosphorylation in AgRP neurons	Yes (multiple non‐synaptic contexts)	(Mishra et al. 2022; Feng et al. 2023; Veeriah et al. 2009; Ortiz et al. 2014)
TrkB (NTRK2)	Ntrk2	P15209	Not mapped (multiple Tyr sites; site(s) unspecified; pY512 tracked as a pathway readout)	Substrate‐trap (inactive D1) capture; TrkB hyper‐Tyr phosphorylation in Ptprd‐null progenitors; overproliferation rescued by TrkB or MEK–ERK inhibition	Neural precursors; restrains MEK–ERK signaling and neurogenesis	No (single primary study)	(Tomita et al. 2020)
PDGFRβ	Pdgfrb	P05622	Not mapped (Tyr sites unspecified; pY1009 tracked as a pathway readout)	Substrate‐trap (inactive D1) capture; PDGFRβ hyper‐Tyr phosphorylation in Ptprd‐null progenitors; overproliferation rescued by MEK–ERK inhibition (cooperates with TrkB)	Neural precursors; cooperates with TrkB to restrain MEK–ERK	No (single primary study)	(Tomita et al. 2020)
Aurora kinase A (AURKA)	Aurka	P97477	Not mapped (pan‐pTyr on AURKA used as readout)	In vitro and cell‐based dephosphorylation; promotes AURKA degradation	Cancer cell contexts (non‐neuronal)	No (single primary study)	(Meehan et al. 2012)

*Note:* For each protein, the table lists the mouse gene symbol and UniProt accession, and in Residue(s) affected reports tyrosine site(s) directly mapped as PTPRD targets; when a PTPRD‐specific site has not been established in the cited context, Not mapped is shown, and the canonical regulatory tyrosine is noted in parentheses for orientation. Evidence type summarizes the strongest orthogonal support (e.g., mapped site, substrate‐trap mutant, biochemical dephosphorylation, genetic or pharmacological pathway rescue), and Primary context/note indicates the biological system. Here, we deliberately do not include putative synaptic substrates inferred solely from phosphotyrosine‐enriched proteomic screens (Park et al. 2020; Henderson et al. 2022; Kim et al. 2025), because individual proteins and/or their specific pTyr sites have not yet been validated by independent biochemical assays, and the three datasets show no overlap in candidate substrates. This keeps Table 4 restricted to targets with direct site information or strong orthogonal evidence.

Unbiased pTyr proteomics is proving invaluable for charting the PTPRD signaling landscape. In *Ptprd* ^−/−^ cortex, mass‐spectrometry profiling detected roughly one hundred synaptic proteins with up‐ and downregulated pTyr levels (Park et al. 2020). A subsequent survey of *Ptprd* ^−/−^ mice produced comparable lists (Henderson et al. 2022), and a recent analysis of *Ptprd‐meB* ^+/−^ mice likewise uncovered extensive pTyr shifts (Kim et al. 2025), implying that disruption of meB‐dependent trans‐synaptic adhesion perturbs phosphorylation networks.

Among the proteins whose pTyr signal increases, which therefore could be direct PTPRD targets, several are presynaptic, including glutamate decarboxylase 1 (67 kDa; *Gad1*), synapsin‐2 (*Syn2*), potassium channel KCNA4 (Kv1.4; *Kcna4*), and Na^+^/K^+^‐ATPase α‐3 (*Atp1a3*) (Kim et al. 2025; Park et al. 2020) as well as synaptotagmin‐1 (*Syt1*), synaptojanin‐1 (*Synj1*), AP‐2 complex subunit β‐1 (βadapt‐in; *Ap2b1*), Caskin‐2 (*Caskin2*) (Henderson et al. 2022). These proteins known to regulate presynaptic functions make them attractive candidates for functional follow‐up. Definitive validation, however, will require additional experiments, including substrate‐trap assays and loss‐of‐phosphorylation rescue experiments.

An interesting notion that emerges from these phosphoproteomic screens is trans‐synaptic signaling across the cleft. In *Ptprd* ^−/−^ mice, both the tyrosine phosphorylation and the postsynaptic enrichment of IL1RAPL1 (the meA‐specific ligand for PTPRD) are reduced (Park et al. 2020). The same pattern is seen for IL1RAP, the meB‐specific ligand, in the meB‐splice‐mutant line (Kim et al. 2025). Mechanistically, two non‐exclusive models can be considered. First, presynaptic PTPRD binds IL1RAPL1/IL1RAP, stabilizing ligand nanoclusters that scaffold postsynaptic kinases (e.g., Src family). Loss of PTPRD declusters ligands, reduces local kinase engagement, and lowers pTyr on ligand tails and adjacent scaffolds, consistent with our phosphoproteomic reductions in IL1RAPL1/IL1RAP pTyr and their enrichment among sites decreased in PTPRD mutants. Second, PTPRD‐dependent dephosphorylation of presynaptic release machinery may alter release probability and NMDAR activation, secondarily modulating postsynaptic tyrosine‐kinase signaling. Reported input‐specific changes in NMDAR content/activation align with this model.

Taken together, the data highlight the dual identity of PTPRD: an adhesion molecule that chooses partners and a phosphatase that modulates their signaling. Fully untangling this complexity will require pinpointing direct D1 substrates, mapping how clustering regulates D1 activity, and determining how each dephosphorylation event feeds back onto synaptic function.

## 9 Non‐Synaptic Functions of PTPRD: Neuronal Genesis, Differentiation, and Migration

PTPRD functions as a molecular brake on embryonic neurogenesis. Two complementary studies converge on the idea that PTPRD restrains embryonic neurogenesis, but they do so from different experimental angles. The first study revealed the phenotype using a constitutive *Ptprd* deletion (*Ptprd* ^−/−^ and *Ptprd* ^+/−^) and acute shRNA/siRNA silencing. They showed that loss of PTPRD (i) expands the pool of Tbr2^+^ intermediate progenitors during mid‐corticogenesis, (ii) yields an enduring surplus of Satb2^+^ and Tbr1^+^ excitatory neurons after birth, and (iii) acts cell‐autonomously; *Ptprd*‐null or *Ptprd*‐siRNA‐treated neural precursors over‐proliferate in neurosphere assays (Tomita et al. 2020). The second study corroborated and extended these findings with a telencephalon‐specific (Emx1‐Cre) conditional KO (Cornejo et al. 2024). Their work confirmed the excess of Tbr2^+^ progenitors and surplus cortical neurons in vivo, thereby ruling out systemic artifacts of the global KO and highlighting the local action of PTPRD within the cortical lineage.

The neurodevelopmental phenotype in *Ptprd* ^+/−^ and *Ptprd* ^−/−^ mice maps to the catalytic D1 phosphatase domain (Tomita et al. 2020). PTPRD normally dephosphorylates TrkB and PDGFRβ; in its absence these receptor tyrosine kinases stay hyperphosphorylated, driving sustained MEK–ERK signaling and unchecked precursor division. Rescue experiments confirm the pathway: pharmacological inhibition of TrkB or MEK restores progenitor numbers in *Ptprd* ^+/−^ and *Ptprd* ^−/−^ cultures. Even heterozygous mutants show a milder yet significant neuronal surplus, indicating haploinsufficiency and offering a cellular explanation for human risk alleles.

The surplus of neurons generated in *Ptprd* ^+/−^ and *Ptprd* ^−/−^ embryos disrupts the next phase of corticogenesis: radial migration and layer formation. Although the normal “inside‐out” birth order is retained, many deep‐layer (Tbr1^+^) and upper‐layer (Satb2^+^) neurons stall below their intended positions, yielding a subtly mis‐laminated yet still six‐layered cortex (Tomita et al. 2020). Two factors probably converge: (i) crowding from the excess cells and (ii) sustained TrkB/PDGFRβ‐driven MEK–ERK signaling, which is known to regulate motility.

PTPRD was appreciated as an axon‐guidance receptor well before its synaptic‐organizer role came to light. Invertebrate genetics first pointed to this function in *C. elegans* (Ackley et al. 2005) and *Drosophila* (Ensslen‐Craig and Brady‐Kalnay 2004; Fox and Zinn 2005; Johnson et al. 2006). In vertebrates, dissociated neuron assays demonstrated that PTPRD acts as a chemotropic guidance cue, directing growth‐cone turning in an attractive manner, and that this effect depends on its phosphatase activity (Sun et al. 2000). Mouse genetics then corroborated the concept: motor‐axon pathfinding in the embryonic spinal cord is impaired when both PTPRD and its close homolog PTPRS are deleted (Uetani et al. 2006), and growth cones from *Ptprd*‐null embryonic dorsal‐root‐ganglion neurons fail to collapse efficiently in response to Sema3A (Nakamura et al. 2017). Collectively, these studies establish Ptprd as a key regulator of axon navigation across phyla.

Nakamura et al. (2017) also showed that the same Sema3A–PTPRD signaling axis orchestrates dendritic patterning. In wild‐type (WT) pyramidal neurons, Sema3A binding to Neuropilin‐1 recruits PTPRD; activation of its D1 domain removes the inhibitory phosphate from Tyr 531 on the Src‐family kinase Fyn. Activated Fyn then phosphorylates downstream effectors such as CRMP2, driving actin‐ and microtubule‐based elongation and branching of basal dendrites. Without PTPRD, cortical and hippocampal pyramidal neurons develop shorter, thinner and sparsely branched arbors, while Fyn remains locked in its Tyr 531‐phosphorylated (inactive) state. Genetic interaction supports the pathway: *Ptprd* ^+/−^; *Fyn* ^+/−^ double heterozygotes display intermediate dendritic defects, indicating that PTPRD is the essential phosphatase linking extracellular Sema3A cues to intracellular Fyn activation.

Taken together, by tempering progenitor proliferation, ensuring orderly laminar migration, guiding axons, and promoting dendritic growth, PTPRD integrates diverse extracellular signals into coherent developmental programs. These non‐synaptic actions provide a unified mechanistic framework for how pathogenic PTPRD variants can lead to neurodevelopmental disorders, connecting excessive neuron production, mis‐laminated cortex, misguided axon pathways, and impoverished dendritic receptive fields.

## 10 Non‐Synaptic Functions of PTPRD: Gliogenesis and Myelination

PTPRD also governs later phases of brain development, guiding the shift from neurogenesis to gliogenesis and pacing the onset of myelination. In a telencephalon‐specific cKO (*Emx1‐Cre*; *Ptprd* ^*fl/fl*^), Cornejo et al. examined embryonic (E16–E18) and early postnatal (P0–P7) cortices and found a marked reduction in glial precursors (Sox9^+^, Olig2^+^) and mature astrocytes and oligodendrocytes (GFAP^+^, Aldh1l1^+^, CC1^+^) (Cornejo et al. 2024). Molecular analyses showed reduced activation of the JAK/STAT pathway (including decreased p‐STAT3) and down‐regulation of STAT3 target genes (e.g., Gfap, Sox9, Aldh1l1). BrdU/EdU pulse‐chase experiments further revealed an extended neurogenic window and delayed gliogenic switch, underscoring PTPRD's role in orchestrating the transition from neurogenesis to gliogenesis.

Zhu et al. showed that *Ptprd* mRNA and protein are transiently upregulated in differentiating oligodendrocytes, peaking between P7 and P15 as CNS myelination accelerates (Zhu et al. 2015). Electron microscopy of the corpus callosum and spinal cord revealed that *Ptprd* ^−/−^ mice display a delayed onset of myelin wrapping, with fewer ensheathed axons and thinner sheaths during the first two postnatal weeks. These early delays were accompanied by reduced expression of myelin proteins such as MBP, PLP1, MAG, and CNP, although node/paranode organization and conduction ultimately normalized. Together, these findings support a “pacing” model in which PTPRD stabilizes early axon–oligodendrocyte contacts to fine‐tune the onset and efficiency of wrapping, while redundancy with PTPRS/PTPRF and activity‐dependent myelin plasticity likely contribute to recovery by adulthood.

## 11 Non‐Synaptic Functions of PTPRD: Metabolism

Beyond its established roles in classical neurodevelopment, recent research has begun to uncover a surprising array of additional non‐synaptic functions for PTPRD, namely hunger and thirst regulations. The canonical hunger circuit involves arcuate nucleus AgRP neurons in the hypothalamus, which represent orexigenic, GABAergic neurons that co‐express NPY and AgRP and project to multiple downstream nodes to acutely regulate food seeking and consumption. Their population activity scales with energy deficit, rising with fasting and ghrelin and falling with adiposity and glycemic signals such as leptin and insulin. At the mechanistic level, for example, the leptin‐receptor–JAK2–STAT3 signaling pathway modulates small‐conductance Ca^2+^‐activated K^+^ (SK) channels that tune AgRP intrinsic excitability and transmitter output.

Recent studies on PTPRD uncovered that, during fasting, white adipocytes release the peptide hormone asprosin into the circulation (Farrag et al. 2022; Duerrschmid et al. 2017; Romere et al. 2016). The hormone traverses the blood–brain barrier and docks onto the extracellular domain of PTPRD that adorns AgRP “hunger” neurons in the arcuate nucleus (Mishra et al. 2022). In AgRP neurons, application of asprosin was shown to reduce STAT3 phosphorylation and to suppress SK channel currents, effects that together increased membrane excitability (Feng et al. 2023; Mishra et al. 2022). Mice lacking PTPRD globally, or selectively in AgRP cells, are lean, hypophagic, and completely unresponsive to exogenous asprosin (Mishra et al. 2022), proving that the phosphatase is the indispensable neural receptor for this adipokine.

Suggesting therapeutic opportunities along the axis, single injections of anti‐asprosin monoclonal antibodies lower food intake, body weight, and blood glucose in diet‐induced obese mice (Mishra et al. 2021). Two receptor‐centric strategies are advancing in parallel. Infusion of a soluble PTPRD ectodomain acts as a decoy sink for circulating asprosin, producing the same appetite‐ and glucose‐lowering effects in vivo (Mishra et al. 2022). Complementing this, newly developed ectodomain antibodies (RD‐43) promote PTPRD dimerization, inhibit its phosphatase activity, and down‐regulate receptor levels, opening a potential receptor‐centric route to appetite control (Qian et al. 2023). These data establish the asprosin–PTPRD pair as a druggable node for metabolic syndrome amenable to both ligand‐ and receptor‐directed interventions, although high‐resolution cryo‐EM or crystallographic confirmation of the asprosin–PTPRD complex is still pending.

For thirst regulation, the lamina terminalis network, containing the subfornical organ (SFO), organum vasculosum of the lamina terminalis (OVLT), and median preoptic nucleus (MnPO) located around the third ventricle of the brain, integrates osmotic and volume cues and drives dipsogenic behavior via hypothalamic and brainstem targets (Augustine et al. 2018; Zhang and Oka 2025; Grove and Knight 2024; Encarnacion‐Rivera et al. 2025). Emerging evidence indicates that cerebellar Purkinje cells contribute to dipsogenic control by shaping the gain and timing of water‐seeking and consummatory actions: selective modulation of Purkinje‐cell ensembles alters drinking, consistent with inhibitory Purkinje output onto deep cerebellar nuclei that, in turn, influence forebrain and parabrachial nodes engaged by thirst circuits (Chen et al. 2023; Mishra et al. 2024; Hwang et al. 2023; Hashimoto et al. 2018; Ryan et al. 2017).

More specifically, a recent study on PTPRD identifies the cerebellum as an asprosin‐sensing center. Mishravet al. demonstrated that cerebellar Purkinje neurons express PTPRD and are robustly excited by circulating asprosin, triggering an immediate increase in water drinking (Mishra et al. 2024). Optogenetic or chemogenetic activation of Purkinje cells mimicked this dipsogenic response, whereas Purkinje‐cell‐specific PTPRD ablation (*L7‐Cre*; *Ptprd* ^fl/fl^) reduced baseline water intake and completely eliminated asprosin‐induced thirst without affecting food consumption or motor learning/coordination. Thus, the same asprosin‐PTPRD ligand–receptor pair independently controls two fundamental homeostatic drives, hunger via hypothalamic AgRP neurons and thirst via cerebellar Purkinje neurons, revealing the cerebellum as an endocrine sensor and positioning the asprosin‐PTPRD axis as a promising target for fluid‐balance disorders.

Together, these studies recast PTPRD from a developmentally important phosphatase to a versatile endocrine receptor that couples peripheral metabolic state to central circuits controlling both energy and hydration homeostasis. Ongoing structural and pharmacological efforts aimed at ligand sequestration, receptor blockade, or allosteric modulation promise translational opportunities across obesity, diabetes, and thirst dysregulation.

## 12 Temporal Dynamics of PTPRD‐Mediated Synaptic and Non‐Synaptic Regulation

**FIGURE 3:**
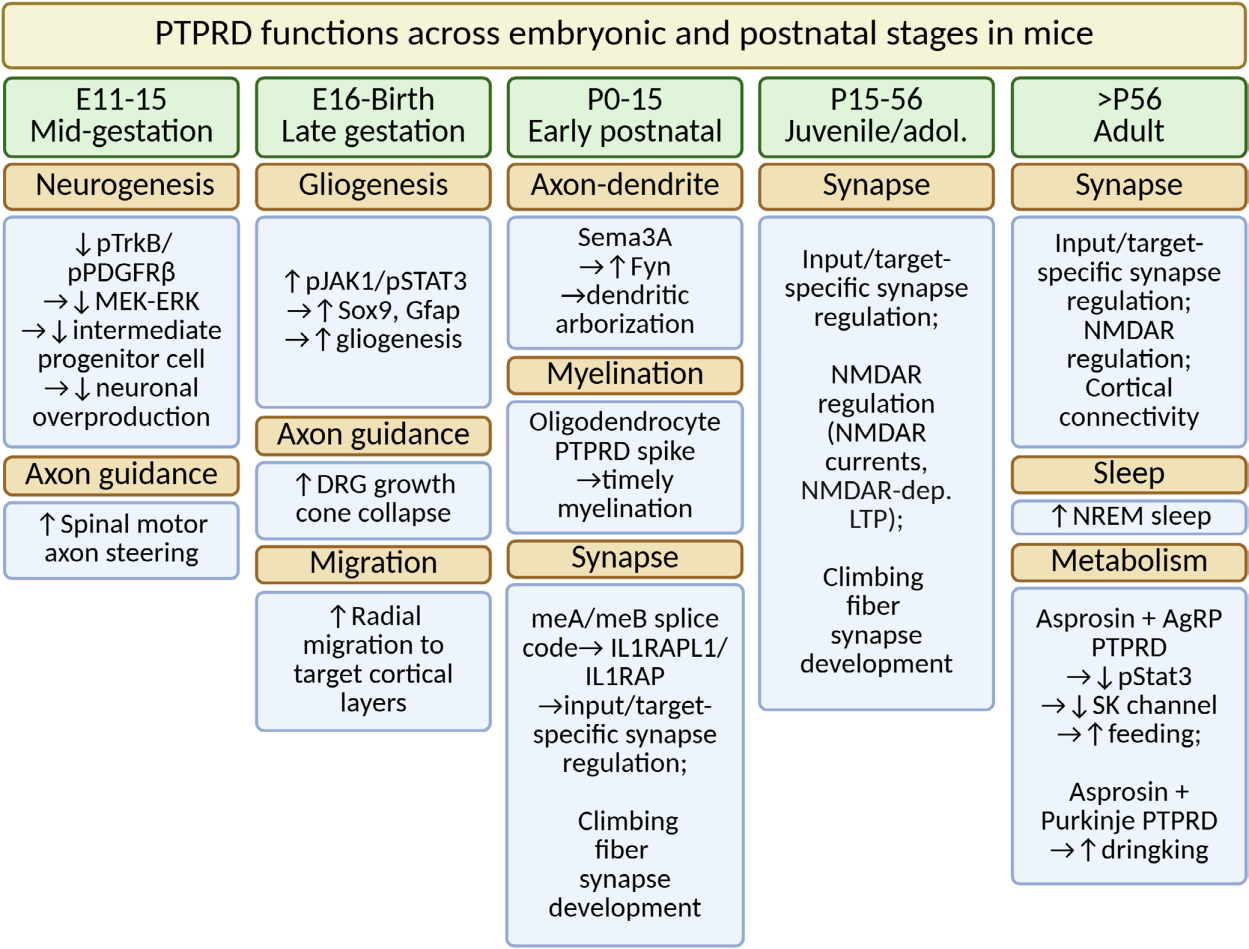
Temporal changes of PTPRD functions from embryogenesis to adulthood in the mouse brain. (A) The diagram arranges experimentally documented roles of PTPRD along five successive developmental windows. Within every window, tan boxes designate the major biological program influenced by PTPRD, and the adjoining blue boxes summarize the key molecular events or phenotypic outcomes. In mid‐gestation (E11–E15), PTPRD is expressed in Pax6^+^ radial glia and Tbr2^+^ intermediate progenitors. De‐phosphorylation of TrkB and PDGFRβ by the D1 domain attenuates MEK–ERK signaling, restrains progenitor expansion, and prevents cortical neuron overproduction. Concomitantly, PTPRD guides spinal motor‐axon trajectories. In late gestation (E16–birth), as neurogenesis gives way to gliogenesis, PTPRD activates the JAK1‐STAT3 pathway, up‐regulates Sox9 and GFAP, and boosts astro‐ and oligodendro‐glial output. It also promotes orderly radial migration and continues to steer spinal and dorsal root ganglion (DRG) axons. During early postnatal stages (P0–P15), semaphorin‐3A engagement triggers PTPRD‐dependent Fyn activation, driving elaboration of basal dendrites. A transient surge of PTPRD in differentiating oligodendrocytes accelerates initial myelination. Splice‐dependent inclusion of mini‐exons meA or meB dictates circuit‐specific trans‐synaptic partnerships (e.g., PTPRD‐IL1RAPL1 vs. PTPRD‐IL1RAP) that establish or stabilize excitatory synapses. During juvenile and adolescent stages (P15–P28 and P28–P56, respectively), presynaptic PTPRD sustains pathway‐ and target‐selective synapse maturation, maintains postsynaptic NMDAR content, supports long‐term potentiation, and is required for proper development of climbing‐fiber synapses on Purkinje cells. In adults (> P56), by balancing excitatory and inhibitory inputs, PTPRD secures medial prefrontal connectivity; loss of function yields hyper‐connected microcircuits, exaggerated hippocampal LTP, and fragmented non‐REM sleep with reduced δ‐power. Systemically, circulating asprosin binds PTPRD on hypothalamic AgRP neurons (driving orexigenic signaling via STAT3 de‐phosphorylation and SK‐channel closure) and on cerebellar Purkinje cells (promoting thirst‐driven drinking). Collectively, the figure illustrates how a single adhesion‐coupled phosphatase integrates extracellular cues with tyrosine‐dephosphorylation signaling to coordinate neurogenesis, gliogenesis, neuronal migration, neurite architecture, myelination, synapse specification, circuit plasticity, sleep regulation, and metabolic homeostasis across the lifespan.

To summarize the non‐synaptic roles of PTPRD across developmental stages, during mid‐gestation (E11–E15), PTPRD regulates cortical neurogenesis: by dephosphorylating TrkB and PDGFRβ it dampens MEK–ERK activity, preventing an over‐expansion of Tbr2^+^ intermediate progenitors and the later surplus of excitatory neurons that appears when *PTPRD* is lost. As development proceeds (E16 to birth), PTPRD is required for normal JAK/STAT activation that promotes gliogenic fate; without it, astro‐ and oligodendro‐glial output falls and the neuron‐to‐glia ratio skews upward. In the early postnatal period PTPRD is transiently up‐regulated in differentiating oligodendrocytes, supporting timely onset of myelin wrapping and sheath growth. It also couples Semaphorin‐3A cues to Fyn activation, ensuring proper growth‐cone collapse and dendritic branching. In adulthood, PTPRD serves as the asprosin receptor on hypothalamic AgRP neurons, linking peripheral energy status to feeding behavior, and also on cerebellar Purkinje cells to link thirst to drinking behavior. Thus, beyond its synaptic duties, PTPRD times neuron production, orchestrates the neuro‐glia hand‐off, shapes neurites, regulates myelin onset and modulates appetite and drinking (Figure 3).

## 13 Perspectives and Future Directions

PTPRD expression is strongly spatiotemporal. Single‐cell and spatial transcriptomic atlases show its progression from ventricular‐zone progenitors to late‐maturing cortical and subcortical neurons, revealing region‐ and stage‐specific transcriptional waves. Whether these mRNA surges translate into parallel shifts in protein abundance remains unresolved. Next‐generation single‐cell proteomic platforms can be used to overlay a high‐resolution proteomic landscape onto the existing transcriptomic framework. Integrating these complementary data sets will be essential for dissecting how transcriptional regulation, post‐transcriptional control, and protein turnover converge to determine the functional availability of PTPRD throughout neurodevelopment and into adulthood.

Finer‐scale analyses are feasible with knock‐in mouse lines carrying tdTomato at the endogenous Ptprd locus, and engineered HaloTag knock‐ins could provide additional flexibility. These models permit direct visualization of PTPRD within nascent axons, dendrites, and pre‐ or postsynaptic compartments across developmental time. Coupling such reporters to super‐resolution microscopy and proximity‐dependent biotinylation will provide the molecular specificity and spatial precision needed to characterize PTPRD in neuronal subregions. Applied longitudinally or under defined sensory, hormonal, or metabolic manipulations, these approaches will reveal how experience and systemic state dynamically regulate the subcellular distribution and function of PTPRD.

Although biochemical studies have confirmed several trans‐synaptic partners of PTPRD, including IL1RAPL1, IL1RAP, Slitrks, and SALM/LRFN isoforms, and the metabolic hormone asprosin, a considerable fraction of its ligand repertoire may remain unidentified. In vivo proximity‐labeling strategies that fuse the endogenous PTPRD ectodomain to APEX2 or TurboID should capture transient extracellular interactors that elude conventional pull‐down assays. High‐content protein‐microarray screens offer an additional, unbiased route to discovery. Candidate ligands emerging from these approaches can then be prioritized *in silico* with structure‐prediction engines such as AlphaFold‐Multimer and subsequently subjected to rigorous biochemical and functional validation.

Alternative splicing of the meA and meB mini‐exons represents a second tier of PTPRD regulation. Inclusion or exclusion of these cassettes subtly remodels the first Ig domain, thereby modulating ligand specificity. While their importance for synaptogenic adhesion is established, their roles in axon guidance, neuronal migration, dendritic morphogenesis, and metabolic circuitry are largely unexplored. CRISPR‐engineered exon swaps between *Ptprd* and its paralogs (*Ptprs*, *Ptprf*), coupled with single‐cell transcriptomics and high‐resolution neuronal morphometry, could elucidate how splice choice reshapes developmental trajectories. Complementary long‐read sequencing of neurons exposed to patterned activity or metabolic stimuli will clarify whether mini‐exon usage is developmentally fixed or subject to dynamic regulation.

At a mechanistic level, the catalytic D1 phosphatase domain serves as a pivotal switch that couples extracellular adhesion to intracellular signaling. High‐resolution structures of full‐length PTPRD in both ligand‐free and ligand‐bound states are needed to delineate the allosteric pathways by which ectodomain engagement modulates D1 activity. Complementary circuit‐resolved phospho‐proteomic screens that deploy catalytically inert “substrate‐trap” mutants (i.e., Cys‐to‐Ser in D1) can distinguish direct D1 targets from secondary phosphorylation changes. Initial datasets nominated Synapsin 2, Kv1.4, SYT1, SYNJ1, and ATP1α3 as candidate substrates, although direct validation is still required.

PTPRD almost certainly does not act in isolation at the synapse. Biochemical evidence shows that presynaptic PTPRD forms *cis* complexes with neurexins and netrin‐G1 (Han et al. 2020; Song et al. 2013). PTPRD interactions with its paralogs PTPRS and PTPRF are plausible but remain untested. Systematic “layered” genetics—single, double, and triple KOs followed by rescue with chimeric receptors that interchange ectodomains, mini‐exon configurations, or liprin‐binding motifs may be required to define when PTPRD performs unique versus redundant functions. In situ proximity‐ligation or split‐TurboID assays can then map the nano‐domains where these *cis* assemblies nucleate and determine whether they scaffold, stabilize, or modulate presynaptic and postsynaptic (NMDAR and AMPAR) nanoclusters.

Beyond its canonical synaptic roles, PTPRD also operates at the interface of metabolism and behavior. The discovery that circulating asprosin engages PTPRD on hypothalamic AgRP neurons and cerebellar Purkinje cells establishes a direct molecular link between systemic energy and hydration states and motivational outputs. Attaining a high‐resolution structure of the asprosin‐PTPRD complex will identify targetable key residues for neutralizing antibodies, decoy peptides, or small molecules capable of modulating feeding and thirst without perturbing broader synaptic functions. In parallel, chemogenetic interrogation of PTPRD‐positive metabolic circuits will determine whether orexigenic and dipsogenic drives diverge downstream of the receptor or converge upon a unified effector pathway.

In summary, PTPRD exemplifies how a single membrane protein can translate finely tuned adhesion codes into intracellular tyrosine‐dephosphorylation cascades. Closing the remaining conceptual gaps will require an integrated strategy combining multi‐omic mapping, context‐specific genetics, and high‐resolution structural biology. Success in these areas could reposition PTPRD from a mechanistic curiosity to a tractable therapeutic target for a spectrum of disorders encompassing neurodevelopment, sleep dysfunction, metabolism, and neurodegeneration.

## Author Contributions

**Eunjoon Kim:** funding acquisition, writing – original draft. **Seoyeong Kim:** writing – original draft. **Jae Jin Shin:** writing – original draft. **Muwon Kang:** writing – original draft. **Yunho Yi:** writing – original draft.

## Conflicts of Interest

The authors declare no conflicts of interest.

## Data Availability

The authors have nothing to report.
